# Migration of Antarctic Minke Whales to the Arctic

**DOI:** 10.1371/journal.pone.0015197

**Published:** 2010-12-22

**Authors:** Kevin A. Glover, Naohisa Kanda, Tore Haug, Luis A. Pastene, Nils Øien, Mutsuo Goto, Bjørghild B. Seliussen, Hans J. Skaug

**Affiliations:** 1 Institute of Marine Research, Bergen, Norway; 2 Institute of Cetacean Research, Tokyo, Japan; 3 Institute of Marine Research, Tromsø, Norway; 4 Department of Mathematics, University of Bergen, Bergen, Norway; Natural History Museum of Denmark, Denmark

## Abstract

The Antarctic minke whale (*Balaenoptera bonaerensis*), and the common minke whale found in the North Atlantic (*Balaenoptera acutorostrata acutorostrata*), undertake synchronized seasonal migrations to feeding areas at their respective poles during spring, and to the tropics in the autumn where they overwinter. Differences in the timing of seasons between hemispheres prevent these species from mixing. Here, based upon analysis of mitochondrial and microsatellite DNA profiles, we report the observation of a single *B. bonaerensis* in 1996, and a hybrid with maternal contribution from *B. bonaerensis* in 2007, in the Arctic Northeast Atlantic. Paternal contribution was not conclusively resolved. This is the first documentation of *B. bonaerensis* north of the tropics, and, the first documentation of hybridization between minke whale species.

## Introduction

In a review of morphological [1] and genetic data [2], [3], minke whales were recently divided into two species [4]; the Antarctic minke whale (*Balaenoptera bonaerensis*) which is confined to the southern hemisphere, and the common minke whale (*B. acutorostrata*) which is cosmopolitan. The common minke whale is further divided into three sub-species; the North Atlantic common minke whale (*B.a. acutorostrata*), the North Pacific common minke whale (*B.a. scammoni*), and the dwarf common minke whale (*B.a.* unnamed sub-species), which is thought to be confined to the southern hemisphere.

A combination of mark-recapture [5], ecological markers [6], [7], and sighting surveys [8] indicate that *B. bonaerensis* undertake seasonal migrations between feeding grounds in the Antarctic waters in the summer (south of 60°S), and breeding grounds in the tropical or temperate regions in the winter. Sighting data from the period 1976–1987 [8] showed that *B. bonaerensis* moved southward from the breeding areas by October-November, and that most of them had migrated into Antarctic waters by January.

*B.a. acutorostrata* occur in the entire North Atlantic during the northern hemisphere summer months, limited in the northern range by the ice [9]. Although their winter distribution is not fully elucidated, they probably migrate to southern latitudes, inhabiting temperate and tropical waters. Sightings have been made as far south as 16°N on the western side [10], 14°N on the eastern side [11] and 10°40′N in the offshore Northeast Atlantic [12]. *B.a. acutorostrata* has been exploited in small-type whaling operations since the 1920s [13]. Since 1996, Norway has maintained an individual based DNA register for *B.a. acutorostrata*. In 1996 (whale 1) and 2007 (whale 2), individual whales deviating from the genetic profile for *B.a. acutorostrata* were captured in the Northeast Atlantic. Size, girth and blubber thickness were similar to *B.a. acutorostrata* [14], but whale 1 lacked the characteristic white patch on the flippers (Table 1). No deviating morphological characteristics where reported for whale 2, although it is not possible to exclude the possibility that this was overlooked at sea. Size of both whales suggests a minimum age of 15 years [15]. Here, we report the identification of these two whales using a combination of genetic data from both mtDNA sequencing and microsatellite DNA fragment analysis.

**Table 1:** Biological records for two atypical minke whales captured in the Northeast Atlantic.

Individual	Position	Biological stats	Comments
Whale 130 June 1996	70°57′N, 8°51′W	Male, length 820 cm, girth 386 cm, blubber 50, 140, 30 mm*	No white patch on flippers. One of six whales captured in the close vicinity within 3 days (29 June–1^st^ July)
Whale 220 June 2007	78°02′N, 11°43′E	Female**, length 825 cm, girth 400 cm, blubber 35, 140, 35 mm*	No abnormalities reported. One of eight whales taken in the close vicinity on the same day.

*Blubber measured dorsal behind blowhole, behind dorsal fin, lateral above flipper, respectively,

**Reproductive status not determined.

## Methods

### Samples

**Figure 1:**
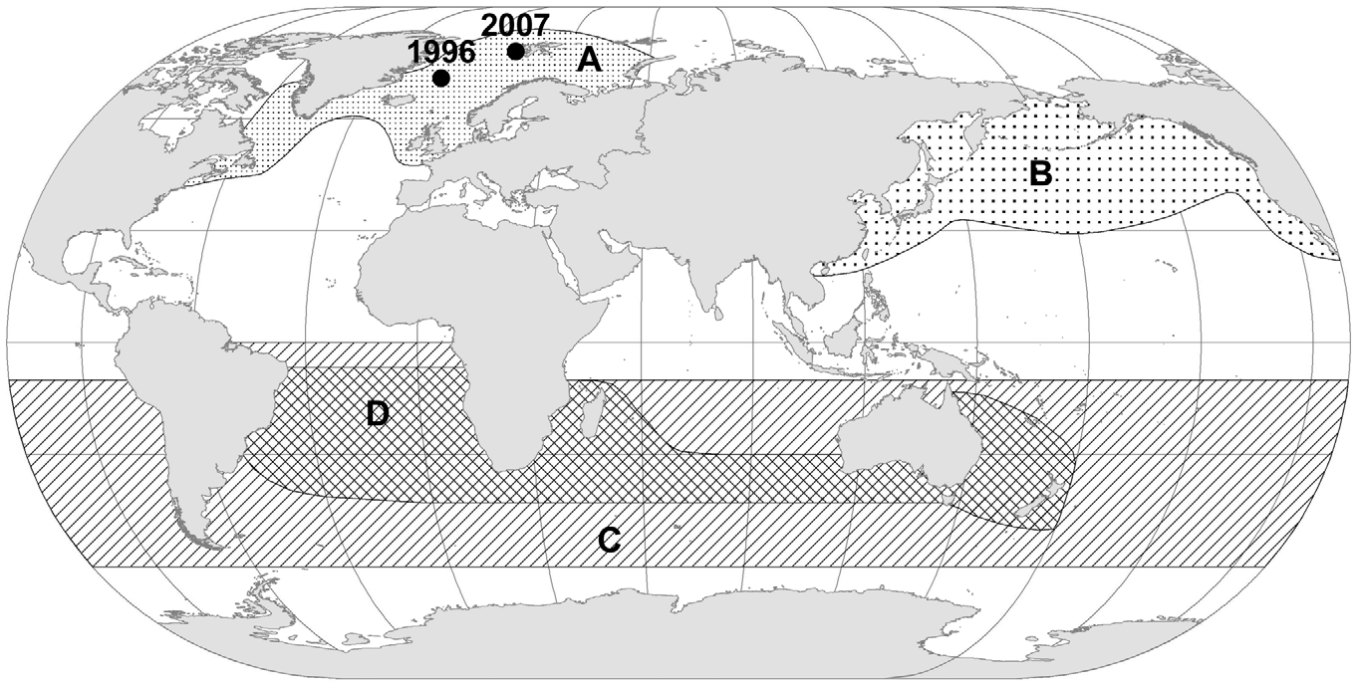
Global distribution of minke whales during northern hemisphere summer feeding season. A: *Balaenoptera a. acutorostrata*, B: *B. a. scammoni*, C: *B. bonaerensis*, D: *B. a.* unnamed subspecies (dwarf minke).1996 and 2007 refers to locations of capture for two atypical whales.

The Norwegian DNA register for *B.a. acutorostrata* consists of 7066 genetic profiles from 7139 individuals captured in the Northeast Atlantic in the period 1996–2008. Individual genetic profiles are produced through a combination of 10 microsatellite loci, sequencing part of the mtDNA control region, and a mysticetes sex marker (Supporting Text S1). In 1996, and 2007, two whales deviating from the typical genetic profile for *B.a. acutorostrata* were captured in the Northeast Atlantic (Fig. 1). In order to identify these two individuals, a mixture of mtDNA (to look at maternal contribution) and microsatellite DNA analyses (to look at both paternal and maternal contribution) were conducted.

Previous studies based on mtDNA [16], [17] have shown fixed differences between *B. bonaerensis* and *B. acutorostrata*, as well as among the sub-species of *B. acutorostrata*. To identify maternal contribution to whales 1 and 2, mtDNA control region sequences from these individuals were compared to published sequences from minke whales worldwide of known species and geographic origin [16],[17]. In contrast to mtDNA, there is no previous study of minke whales worldwide based on microsatellites. Consequently, in order to identify both paternal and maternal contribution to whales 1 and 2, microsatellite analyses were conducted on samples of *B. bonaerensis* and sub-species of *B. acutorostrata* from different ocean basins. These included 91 *B. bonaerensis* (Antarctic 2004), 91 *B.a. acutorostrata* (Northeast Atlantic 2007), and 95 *B.a. scammoni* (Northwest Pacific 2006). All whale samples, including those from the Norwegian minke whale DNA register and the Japanese whale research programs under special permit in both the western North Pacific and Antarctic, existed prior to this study.

### Laboratory analyses

MtDNA sequencing of whales 1 and 2 was conducted at the Institute of Marine Research by amplifying DNA and thereafter sequencing the PCR product in both the forward and reverse direction. The PCR conditions for the two directions were identical except for the primers: (MT4(M13F) and MT3(M13Rev) (modified from [18]) for the forward PCR product, and BP15851(M13F) (modified from [19]) and MN312(M13R) (Modified from [20]) for the reverse PCR product. All PCR reactions were performed with GoTaqFlexi DNA polymerase (Promega). The amplicon was sequenced by a standard Big Dye Terminator 3.1 protocol (Applied Biosystems) and M13F for forward PCR product and M13R for the reverse PCR product. Sequencing primers and full amplification conditions are presented in Supplementary Text S1.

A total of 13 microsatellites and a sex marker were amplified for all species/sub-species samples including whales 1 and 2. Ten of the microsatellites and the sex marker are routinely used in the Norwegian DNA register for *B.a. acutorostrata* (PCR 1-3), while the remaining three microsatellites (PCR 4) were chosen specifically for this study based upon the fact that they display high or very high F_ST_ values (and therefore provide diagnostic identifications) between minke whale species. The exact amplifications conditions for PCR amplification are presented in Supplementary Text S1. Markers were arranged in four polymerase chain reaction (PCR) multiplexes: PCR 1 =  *GT509* [21], *GATA098* [22], *EV001Pm* [23], *EV037Mn* [23], *GT310* [21]; PCR 2 =  *GT211* [21], *GT575* [21], sex marker [24]; PCR 3 =  *GATA417* [22], *GATA028* [*22*], *GT023* [21]; PCR 4 =  *DIrFCB14* [25], *EV104Mn* [23], *EV94Mn* [23]. PCR fragments were separated and sized in a capillary based ABI 3730XL genetic analyser. Genotypes were first automatically called, then, manually checked by two persons before exporting data. All samples were genotyped twice, and poorly amplified individuals removed from the data set. Whale 1 and 2 were genotyped up to 10 times for each marker on two separate DNA isolations (no inconsistencies were observed between multiple genotyping). Following genotyping, the microsatellites *GT310* and *GATA098* were excluded from the study due to unreliable binning of alleles and poor PCR amplification respectively.

### Statistical analyses

MtDNA sequences from whales 1 and 2 were aligned to sequences of *B. bonaerensis* and sub-species of *B. acutorostrata* [17]. The genealogy of the mtDNA haplotypes was estimated using the Neighbor-Joining method [26] as implemented in the program PHYLIP. Genetic distances among haplotypes were estimated using the program DNADIST of PHYLIP, based on Kimura-2-parameter model. A transition-transversion ratio of 5∶1 was used. The genealogy was rooted using the homologous sequence from nine baleen whale species [18]. To estimate support for each node, a total of 1,000 bootstrap simulations were conducted and the majority-rule consensus genealogy estimated.

In order to characterize the minke whale species/sub-species using the microsatellite data generated here, population genetic summary statistics, F_ST_ values, and potential deviations from Hardy Weinberg equilibrium (HWE) were computed in the programs MSA [27] and Genepop [28]. Following initial characterization of the species/sub-species, data from all 11 microsatellite loci, and a reduced set consisting of 8 loci (see results) was used to perform genetic identification of whales 1 and 2. Identifications based upon microsatellite data were conducted in two programs that use different and complimentary analytical approaches.

The first identification of whales 1 and 2 was performed by Bayesian cluster analysis as implemented in the program Structure [29], [30]. This program was run by using an admixture model, no population prior for all individuals, and the burn-in set to 100 000 MCMC steps, followed by a further 250 000 steps. This was conducted for numbers of populations (*K*) set to 3 (i.e., the number of baseline species/sub-species samples) and 4 (to investigate whether any cryptic structure existed within any of the species/sub-species samples which may assist in the identification of whales 1 and 2), each with three iterations (to check for consistency). In addition to identification of whales 1 and 2 by Bayesian cluster analysis, genetic assignment was conducted in the program GeneClass2[31]. Prior to identification of these two whales however, simulations with the baseline data (i.e., the three species/sub-species samples in addition to 90 F1 hybrids generated in HYBRIDLAB1.0 [32] between *B.a. acutorostrata* and *B. bonaerensis*, and *B.a. scammoni* and *B. bonaerensis*) using the self-assignment and leave one out approach was implemented in the GeneClass2. The self-assignment tests give an estimation of the level of accuracy expected from the assignment tests. Following self-assignment simulations, identification of whales 1 and 2 were conducted by using the direct assignment approach. Direct assignment places the individual(s) to be identified in the genetically most similar baseline sample, irrespective of absolute level of similarity. In addition, the probability of excluding each of the two whales from each of the baseline samples in turn was calculated. Exclusion was conducted by Monte-Carlo re-sampling of the baseline with 1000 individuals [33]. All GeneClass2 analyses were in re-computed for whale 2 after having changed the genotype at two loci due to potential genotyping irregularities at these loci (Supplementary Table S1).

## Results and Discussion

### MtDNA analyses

The final data set (whales 1 and 2 and minke whales worldwide examined in [17]) included the first 287 nucleotides of the mtDNA control region. In this data set the sequences of whales 1 and 2 represented singletons. MtDNA sequences for whales 1 (HQ162497) and 2 (HQ162498) have been deposited in the Genbank.

The Kimura -two-parameter distance between whale 1 and the sequences of *B. bonaerensis* in [17] averaged 0.0104. The distance ranged from 0.0730 and 0.0808 when whale 1 was compared with sequences of the sub-species of *B. acutorostrata* in [17]. The distance between whale 2 and the sequences of *B. bonaerensis* averaged 0.0286. The distance ranged from 0.0806 and 0.0865 when whale 2 was compared with sequences of the sub-species of *B. acutorostrata*. In the neighbour-joining based genealogy, sequences of whales 1 and 2 clustered within the *B. bonaerensis* clade with high bootstrap values. In a comparison with a larger data set of *B. bonaerensis* mtDNA sequences (n = 1165, data not published) whale 1 matched the haplotype of 4 individuals while whale 2 was represented by a singleton.

### Microsatellite DNA analyses

Summary statistics for the 11 microsatellite loci are presented (Table 2). Deviations from HWE were observed in all three species/sub-species, however, at the more stringent significance level (α0.001), only the deviations observed in the sample of *B. bonaerensis* remained significant. Some statistical genetic tests assume that the data used adhere to a given set of conditions, for example, that markers are in HWE. Although it has been demonstrated that minor and even serious violations of the assumptions for genetic assignment tests do not necessarily bias the result [34], [35], [36], [37], subsequent analyses were conducted with all 11 loci, in addition to a sub-set of 8 loci (excluding *GT509*, *GT575* and *EV037Mn*).

**Table 2:** Genetic variation within (allelic variation) and among (F_ST_ values) three species/sub-species of minke whales based upon the analysis of 11 microsatellite loci.

Species	N	Locus											Loci pooled
		1	2	3	4	5	6	7	8	9	10	11	A_T_
Allelic variation													
Atl	91	2	2*	3	12	7	12	9	9	11	12	8	87
Pac	95	5	3	5	19	11*	15	13	9	12	8	13*	113
Ant	91	4	16	17	11	20**	37**	16	18**	39	47	16	241
Total	277	9	16	19	28	22	39	18	18	44	51	16	280
He		0.36	0.57	0.48	0.83	0.75	0.88	0.85	0.84	0.87	0.86	0.84	
F_ST_ values													
Atl x Pac		0.038	0.029	0.404	0.099	0.325	0.091	0.045	0.057	0.009	0.086	0.105	0.128
Atl x Ant		0.640	0.248	0.600	0.171	0.152	0.073	0.027	0.068	0.087	0.073	0.130	0.211
Pac x Ant		0.608	0.176	0.430	0.151	0.137	0.050	0.023	0.046	0.062	0.129	0.019	0.171
Global F_ST_		0.537	0.175	0.481	0.141	0.216	0.072	0.032	0.057	0.054	0.097	0.085	0.172
Global (P value)		<0.0001	<0.0001	<0.0001	<0.0001	<0.0001	<0.0001	<0.0001	<0.0001	<0.0001	<0.0001	<0.0001	<0.0001

Locus 1 =  *DIrFCB14*, 2 =  *EV104 Mn*, 3 =  *EV94 Mn*, 4 =  *EV001 Pm*, 5 = *EV037 Mn*, 6 =  *GT509*, 7 =  *GT211*, 8 =  *GT575*, 9 =  *GATA028*, 10 =  *GATA417*, 11 =  *GT023*.

* =  significant from HWE at 0.05,

** =  significant deviation from HWE at 0.001.

Atl  =  *B.a. acutorostrata*, Pac  =  *B.a. scammoni*, Ant  =  *B. bonaerensis*. A_T_  =  total number of alleles, He  =  expected heterozygosity for each locus.

**Figure 2:**
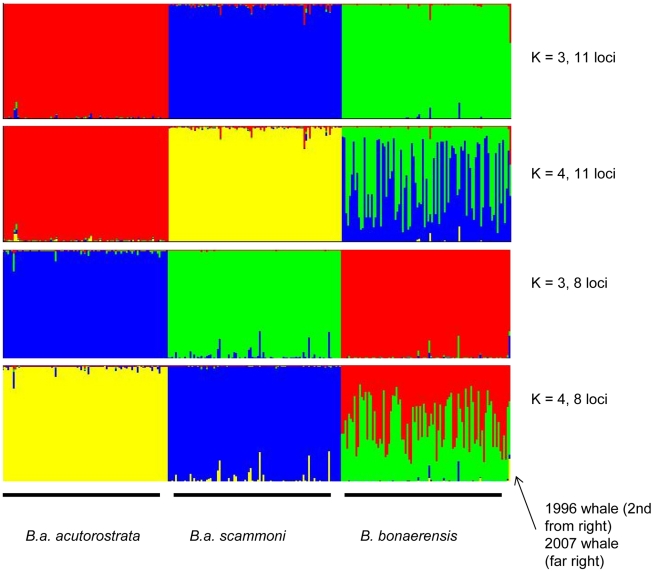
Bayesian cluster analysis identifying two atypical minke whales captured in the Northeast Atlantic. Each vertical line represents a single individual (which can be admixed), and each colour a genetic cluster. Note that colour scheme is not universal between the four runs presented.

Data from the microsatellite markers revealed highly significant genetic differentiation among the three species/sub-species (Table 2). When pooling data from all 11 loci, *B. bonaerensis* was the most genetically distinct. Although these comparisons need to be treated with caution, due to the fact that some of the markers were deliberately chosen to provide the greatest possible diagnostic power for identification of whales 1 and 2, these data concord with the pattern of relatedness among these species/sub-species from previous studies of minke whales using mtDNA [2], [3], [16], [17]. The genetic distinctiveness of the three species/sub-species samples was confirmed through Bayesian clustering analysis (Fig. 2). In addition, the sample of *B. bonaerensis* displayed considerable genetic sub-structure when the number of clusters was set to 4.

The level of genetic differentiation displayed by many of the microsatellites in the present study was very large (seven displayed a pair-wise F_ST_ value over 0.1, four displayed a value over 0.2, and two displayed a value over 0.6). The ability to accurately perform genetic assignment depends upon several factors including the level of genetic differentiation among the baseline (potential source) samples, the number of loci included in the analyses, in addition to sample sizes [34], [35], [38], [39]. The sample sizes, number or loci and level of genetic differentiation in the present data set indicates very good opportunity for genetic identification according to simulations of optimal combinations [38]. Furthermore, this was demonstrated by the self-assignment simulations conducted here. All three species/sub-species samples revealed 100% self-assignment accuracy (i.e., all of the individuals were correctly classified back to their source species/sub-species) when using both 11 and 8 loci. When simulated hybrids were incorporated into the computations (thus increasing baseline sources to 5), accuracy of self-assignment remained very high (11 loci  = 98%; 8 loci  = 96%), demonstrating the suitability of the suite of microsatellite markers to perform identifications of whale 1 and 2, to both species/sub-species and F1 hybrid combinations.

Using data from both 11 and the reduced set of 8 microsatellite loci, whale 1 was diagnostically identified as a pure *B. bonaerensis* (Table 3; Fig. 2; Supplementary Table S1), confirming this individual's migration from the Antarctic to the Arctic. This represents the first documentation of this species north of the tropics. A combination of Bayesian cluster analysis (Fig. 2), genetic assignment (Table 3) and genotype break-down (Supplementary Table S1) rejected whale 2 as pure *B. bonaerensis*. When combined with the fact that the mtDNA haplotype for this whale was *B. bonaerensis*, these data represent the first documentation of a hybrid between a minke whale species. The source, and proportion of *B. acutorostrata* sub-species contribution to this individual was not conclusively resolved however. Examination of available data (Naohisa Kanda unpublished) for nine dwarf minke whales (*B. acutorostrata* unnamed sub-species) genotyped for six markers overlapping with the panel implemented in the present study did not shed further light on this identification. Consequently, while it is not possible to conclusively identify the paternal contribution to whale 2, nor exclude it from being a F1-F*x* hybrid between *B. bonaerensis* and any of the *B. acutorostrata* sub-species, results from Bayesian cluster analysis (Fig. 2) and direct genetic assignment (Table 3) indicate the most likely paternal contribution from *B.a. acutorostrata*.

**Table 3:** Identification of two atypical minke whales captured in the Northeast Atlantic based upon exclusion (i.e., probability) and direct assignment (i.e., closest match).

Individual	Loci	Probability of false exclusion from baseline sample					Direct assignment
		Atl	Pac	Ant	Atl x Ant	Pac X Ant	
Whale 1	11	<0.001	<0.001	0.209	0.003	0.001	Ant
	8	<0.001	<0.001	0.553	0.073	0.112	Ant
Whale 2	11	<0.001	<0.001	<0.001	<0.001	<0.001	Atl X Ant
	8	<0.001	<0.001	<0.001	<0.001	<0.001	Atl X Ant
Whale 2*	11	<0.001	<0.001	<0.001	0.016	0.015	Atl X Ant
	8	<0.001	<0.001	<0.001	0.038	0.039	Atl X Ant

Atl  =  *B.a. acutorostrata*, Pac  =  *B.a. scammoni*, Ant  =  *B. bonaerensis*, Atl x Ant and Pac x Ant  =  simulated F1 hybrids. Whale 2*  =  individuals genotype changed for two loci according to potential genotyping irregularities (Supplementary Table 1). Loci refers to number of microsatellite DNA loci included in the statistical analyses.

### General discussion

Whilst inter-species hybridization has been previously observed between blue (*B. musculus*) and fin (*B. physalus*) whales [40], our study presents the first example of hybridisation between minke whale species. Furthermore, accepting paternal contribution from *B. a. acutorostrata* as most likely, based upon the present analyses, and the fact that whale 1, a pure breeding migrant, was observed in the Northeast Atlantic approximately a decade before the hybrid was reported in the same region, whale 2 represents the first example of a hybrid between two whale species from separate hemispheres. The two alternative paternal contributions to this hybrid, i.e., fathered by *B.a. scammoni* and migrated from the North Pacific to the North Atlantic, or fathered by the *B.a.*subs. (dwarf minke whale) found in the southern hemisphere migrating to the Northeast Atlantic, represent equally unique and dramatic results from an ecological perspective.

Although whales display considerable potential to undertake long migrations, inter-oceanic migrations outside the species boundaries are extremely rare, and previously reported only for the humpback whale within the same hemisphere (*Megaptera novaeangliae*) [41]. It is very possible that the migrations documented here were random events, which may or may not represent a scouting behavior having occurred over many years. Indeed, the Norwegian minke whale DNA register, which provides the unique opportunity to document this infrequent behavior, only goes back as far as 1996. Coincidently, this is the same year that the pure *B. bonaerensis* individual (whale 1) was captured in the Northeast Atlantic. Assuming a total population abundance of 107000 *B.a. acutorostrata* in the Northeast Atlantic [42] a pro-rata estimate of the number of individuals with *B. bonaerensis* mtDNA haplotypes present in this region is 30 (95% CI: 4-107). Consequently, the Northeast Atlantic does not appear to be a major destination of *B. bonaerensis* migration outside its previously documented distribution.

## Supporting Information

Text S1 Extended materials and methods for genotyping conditions.
(DOC)

Table S1 Presence of alleles for two atypical whales in the genetic baseline.
(DOC)
