# Supplementary material for: Migration of Antarctic Minke Whales to the Arctic
Source: PLoS One. 2010 Dec 22;5(12):e15197. doi: 10.1371/journal.pone.0015197 (PMC3008685; doi:10.1371/journal.pone.0015197)
Supplement: Text S1 — Extended materials and methods for genotyping conditions. (DOC) [file pone.0015197.s001.doc]

Supplementary Text S1

Genotyping conditions for microsatellite markers

A total of 13 microsatellites and a sex marker were analyzed for all individuals according to the below conditions. Amplifications were conducted in four multiplex reactions (PCR 1-4), and in all cases, the 5´ dye labels were supplied by Applied Biosystems Inc (ABI). All PCR reactions contained 1 unit Go Taq polymerase (Promega) with 1x associated buffer (working out at 1.5-2 mM MgCL_2_ per reaction). All PCR amplifications were based on a 2 minute heat start at 94°C, denaturizing for 20 seconds at 94°C, annealing for 45 seconds, elongation at 72°C for 1 minute and a final hold at 4°C.

PCR 1: 1.7µM (6-FAM) primer *GT509* [1], 0.3 µM (6-FAM) primer *GATA098* [2], 0.15 µM (6-FAM) primer *EV001Pm* [3], 0.17µM (VIC) primer *EV037Mn* [3], 0.18 µM (NED) primer *GT310* [1], 1.5 mM MgCl_2_ and 0.2 mM dNTP, 28 amplification cycles with annealing temperature of 59°C.

PCR 2: 0.2 µM (NED) primer *GT211* [1], 0.4 µM (NED) primer *GT575* [1], 0.1 µM (PET) primer *ZFYX0582F*, 0.25 µM primer *ZFY0752R*, 0.2 µM primer *ZFX0785R* [4], 2 mM MgCl_2_ and 0.2 mM dNTP, 30 amplification cycles with annealing temperature of 59°C.

PCR 3: 0.07µM (NED) primer *GATA417*[2], 0.25µM (VIC) primer *GATA028[2]*, 0.08µM (VIC) primer *GT023* [1], 2 mM MgCl_2_ and 0.2 mM dNTP, 28 amplification cycles with annealing temperature of 54ºC.

PCR 4: 0.2µM (6-FAM) primer *DIrFCB14* [5], 0.2 µM (NED) primer *EV104Mn* [3], 0.2 µM (PET) primer *EV94Mn* [3], 1.5 mM MgCl_2_ and 0.2 mM dNTP, 29 amplification cycles with annealing temperature of 59ºC.

DNA fragments were separated and sized in a capillary based ABI 3730 genetic analyzer. Genotypes were first automatically called, then manually checked by two persons before exporting data. All samples were genotyped twice, and poorly amplified individuals removed. Whale 1 and 2 were genotyped up to 10 times for each marker on two separate DNA isolations. Markers *GT310* and *GATA098* were excluded from the analyses due to unreliable binning of alleles and PCR amplification respectively.

Sequencing the mtDNA control region

All mtDNA sequencing performed at the Institute of Marine Research in Bergen were conducted according to the below conditions, whereas sequencing for the other samples has been described previously [6].

MtDNA sequencing of the samples from the Atlantic was performed by amplifying DNA and thereby sequencing the PCR product in both the forward and reverse directions. The PCR conditions for the two directions were identical except for the primers, and each reaction contained 0.5 units Go Taq polymerase (Promega) with 1x associated buffer (working out at 1.5 mM MgCL_2_), and 0.2 mM dNTP. Primer concentrations were 0.2 µM of each primer (MT4(M13F) and MT3(M13Rev) (modified from [7]) for the forward PCR product, and BP15851(M13F) (modified from [8]) and MN312(M13R) (Modified from [9]) for the reverse PCR product. Amplification was as follows: hot start at 94°C for 2 min, followed by 30 cycles of denaturizing at 94°C for 50 seconds annealing at 53°C for 50 seconds and elongation at 72°C for 3min 30 seconds, and finally a 10 min elongation at 72°C and a 4°C hold. The amplicon was sequenced by a standard Big Dye 3.1 protocol (Applied Biosystems) and M13F for forward PCR product and M13R for the reverse PCR product. Sequencing primers:

MT4(M13F) - GTAAAACGACGGCCAGTCCTCCCTAAGACTCAAGGAAG

MT3(M13Rev)- CAGGAAACAGCTATGACCCATCTAGACATTTTCAGTG

BP15851(M13F) -GTAAAACGACGGCCAGTGAAGAAGTATTACACTCCACCAT

MN312(M13R) - CAGGAAACAGCTATGACCCGTGATCTAATGGAGCGGCCA

M13F - GTAAAACGACGGCCAGT

M13R – CAGGAAACAGCTATGACC

1. Berube M, Jorgensen H, McEwing R, Palsboll PJ (2000) Polymorphic di-nucleotide microsatellite loci isolated from the humpback whale, Megaptera novaeangliae. Molecular Ecology 9: 2181-2183.

2. Palsbøll P, Bérubé M, Larsen AH, Jørgensen H (1997) Primers for the amplification of tri- and tetramer microsatellite loci in cetaceans. Molecular Ecology 6: 893-895.

3. Valsecchi E, Amos W (1996) Microsatellite markers for the study of cetacean populations. Molecular Ecology 5: 151-156.

4. Palsbøll PJ, Clapham PJ, Jørgensen H, Larsen H, Mattila DK, et al. (1998) The value of parallel analysis of uni- and bi-parental inherited loci: the North Atlantic humpback whale (Megaptera novaeangliae). In: Karp, A, Isaac, PG, Ingram, DS (eds) Molecular tool for screening biodiversity: plants and animals, London: Chapman and Hall, 426-430.

5. Buchanan FC, Friesen MK, Littlejohn RP, Clayton JW (1996) Microsatellites from the beluga whale Delphinapterus leucas. Molecular Ecology 5: 571-575.

6. Pastene LA, Goto M, Kanda N, Zerbini AN, Kerem D, et al. (2007) Radiation and speciation of pelagic organisms during periods of global warming: the case of the common minke whale, Balaenoptera acutorostrata. Molecular Ecology 16: 1481-1495.

7. Arnason U, Gullberg A, Widegren B (1993) Cetacean mitochondrial DNA control region sequences of all extant baleen whales and 2 sperm whale species. Molecular Biology and Evolution 10: 960-970.

8. Larsen AH, Sigurjonsson J, Oien N, Vikingsson G, Palsboll P (1996) Populations genetic analysis of nuclear and mitochondrial loci in skin biopsies collected from central and Northeastern North Atlantic humpback whales (Megaptera novaeangliae): Population identity and migratory destinations. Proceedings of the Royal Society of London Series B-Biological Sciences 263: 1611-1618.

9. Palsboll PJ, Clapham PJ, Mattila DK, Larsen F, Sears R, et al. (1995) Distribution of mtDNA haplotypes in North-Atlantic humpback whales – the influence of behaviour on population structure. Marine Ecology-Progress Series 116: 1-10.
