# Supplementary material for: Migration of Antarctic Minke Whales to the Arctic
Source: PLoS One. 2010 Dec 22;5(12):e15197. doi: 10.1371/journal.pone.0015197 (PMC3008685; doi:10.1371/journal.pone.0015197)
Supplement: Table S1 — Presence of alleles for two atypical whales in the genetic baseline. (DOC) [file pone.0015197.s002.doc]

| Table S1. Presence of alleles for two atypical whales in the genetic baseline. | | | | | | |  |  |
| --- | --- | --- | --- | --- | --- | --- | --- | --- |
|  |  |  |  |  |  |  |  |  |
| Marker (genotype) | | Whale 1 (1996) | |  | Marker (genotype) | | Whale 2 (2007) |  |
|  |  |  |  |  |  |  |  |  |
|  | *B.a. acutorostrata* | *B.a. scammoni* | *B. bonaerensis* | |  | *B.a. acutorostrata* | *B.a. scammoni* | *B. bonaerensis* |
| DIrFCB14 |  |  |  |  | DIrFCB14 |  |  |  |
| 266 |  |  | x |  | 258 | x | x |  |
| 266 |  |  | x |  | 270 |  |  | x |
| EV104 |  |  |  |  | EV104 |  |  |  |
| 147 | x | x | x |  | 143 |  |  | x |
| 149 | x | x | x |  | 149 | x | x | x |
| EV94 |  |  |  |  | EV94 |  |  |  |
| 195 |  |  | x |  | 195 |  |  | x |
| 195 |  |  | x |  | 211 | x | x | x |
| EV001 |  |  |  |  | EV001 |  |  |  |
| 134 |  | x | x |  | 128 |  |  | x |
| 136 |  | x | x |  | 151 | x | x |  |
| EV37 |  |  |  |  | EV37 |  |  |  |
| 203 | x | x | x |  | 203 | x | x | x |
| 203 | x | x | x |  | 203 | x | x | x |
| GT211 |  |  |  |  | GT211 |  |  |  |
| 104 | x | x | x |  | 100 |  | x | x |
| 110 | x | x | x |  | 106 | x | x | x |
| GT509 |  |  |  |  | GT509 |  |  |  |
| 174 |  |  | x |  | 190 |  |  | x |
| 190 |  |  | x |  | 207 | x | x | x |
| GT575 |  |  |  |  | GT575 |  |  |  |
| 146 |  |  | x |  | 134 |  |  | x |
| 146 |  |  | x |  | 154 | x | x | x |
| GATA028 |  |  |  |  | GATA028(*1) |  |  |  |
| 176 |  |  | x |  | 231 |  |  | x |
| 211 | x | x | x |  | 231 |  |  | x |
| GATA417 |  |  |  |  | GATA417(*2) |  |  |  |
| 238 |  |  | x |  | 212 |  |  |  |
| 254 |  |  | x |  | 236 | x |  | x |
| GTO23 |  |  |  |  | GTO23 |  |  |  |
| 105 | x | x | x |  | 103 | x | x | x |
| 105 | x | x | x |  | 111 | x | x | x |
| X denotes allele observed in genetic baseline. | | |  |  |  |  |  |  |
| (*1) = very weak but consistent peak detected at 207. 207 observed in all baseline populations. | | | | | | |  |  |
| (*2) = 212 not observed in any sample, when placed into closest bin (211 or 213), both alternative alleles observed in all samples. | | | | | | | | |
